# Detroit Interventional Pain Assessment Scale: A Pain Score and Method for Measuring and Evaluating Post-Operative Pain Management—A Prospective Study

**DOI:** 10.3390/medicina59111976

**Published:** 2023-11-09

**Authors:** Lauryn J. Boggs, Sasha A. Stine, Barbara J. Boggs-Hughey, Andreea Geamanu, Bryan E. Little, Hussein F. Darwiche, Rahul Vaidya

**Affiliations:** 1 Department of Orthopedic Surgery, Wayne State University, Detroit, MI 48202, USA; 2 Department of Orthopedics and Sports Medicine, Wayne State University, Detroit, MI 48202, USA

**Keywords:** post-operative pain management, pain assessment tools, orthopedic surgery, opioids, morphine milligram equivalent

## Abstract

*Background and Objectives*: Orthopedic surgeons commonly prescribe opioids, surpassing all medical specialties. Our objective was to develop a pain management scale that captures medication use, patient-reported pain scores, and helps orthopedic surgeons evaluate their post-operative prescribing practice. *Materials and Methods*: An IRB-approved prospective study followed 502 post-operative orthopedic surgery patients over a six-month period. All patients were surveyed in an orthopedic clinic at a Level 1 US Trauma Center, during a routine follow-up. Patient pain satisfaction was assessed using the validated Interventional Pain Assessment (IPA) scale, which uses three categories: 0 (no pain), 1 (tolerable pain), and 2 (intolerable pain). Daily narcotic use was translated to morphine milligram equivalents (MMEs) using the Michigan Automated Prescription System (MAPS) narcotics registry. When patient pain satisfaction and narcotic usage were combined, this scale was called the Detroit Interventional Pain Assessment (DIPA) scale. *Results*: The five classes based on common prescription and usage of narcotics in this cohort include the following: A (no pain medication), B (over-the-counter medication), C (occasional use of short-acting narcotics 1–30 MMEs), D (consistent/regular use of short-acting narcotics 31–79 MMEs), and E (long-duration or stronger short-acting narcotics 80+ MMEs). Patients were most satisfied with their pain management at six weeks (80.5%) and three months (75.65%), and least satisfied at two weeks (62.5%) and six months (60.9%). Additional information displayed on the DIPA graph revealed there was a significant decrease in the percentage of patients on narcotics at two weeks (65.2%) to six months (32.6%) at *p* < 0.001. *Conclusions*: The DIPA pain scale shows the relationship between patient pain perception and opioid prescription/usage, while also tracking prescriber tendencies. Providers were able to visualize their post-operative pain management progression at each designated clinic visit with corresponding alphabetical daily MME categories. In this study, results suggest that surgeons were not effective at managing the pain of patients at two weeks post-operative, which is attributed to an inadequate number of pain pills prescribed upon discharge. Overall, the DIPA graph signaled that better pain management interventions are necessitated in periods with lower efficiency scores.

## 1. Introduction

The opioid crisis has become endemic to the United States since its inception two decades ago, and opioid overuse is a major problem for orthopedic providers [1]. A recent study involving 81,459 patients revealed that, out of all patient prescribed narcotics, orthopedic surgeons prescribed opioids in 41.76% of cases, surpassing all medical specialties [2]. On average, it takes three months to recover from an orthopedic surgery [3]. Opioids taken consistently during this time correspond to the same amount of time for one to become a regular opioid user [4]. Providers must be cognizant of their prescription patterns to avoid complications of opioid overuse [5,6,7,8].

The Interventional Pain Assessment (IPA) scale was developed using only three descriptive categories: no pain, tolerable pain, and intolerable pain [9]. The IPA was validated against the Numerical Rating Scale (NRS)—the 0 to 10 pain scale—and, unlike the NRS, it was designed to provide a treatment direction based on the pain perception of patients [9,10,11,12]. Patients who report no pain or tolerable pain are satisfied with their pain management, and those who report intolerable pain need an intervention in their regimen. When compared to the common NRS, 82% of patients preferred the IPA because of its simplicity, its sensitivity to changes in patients’ pain experience, and its guidance during treatment [9].

Given an increase in narcotic abuse, the CDC has taken precautions to increase awareness of opioid addiction by using morphine milligram equivalents (MMEs) [12]. This number provides a way for prescribers to compare the various formulations of opioids based on their morphine equivalents [13]. However, while an MME value can provide physicians with quantifiable numbers, the numbers are difficult to interpret because there are no categories and only vague cutoffs. The CDC cautions against daily MMEs greater than 50 and warns of daily MME values over 90 due to the increased probability of respiratory distress [13,14,15]. While these two numbers provide physicians with rough guidelines, there is no way of knowing which opioids and which scheduling classification correspond to these guidelines. The goal was to make a pain scale reflecting the pain medications taken by the patient, specifically in the post-operative orthopedic population. The second aim was to develop a visual representation of patients’ pain scales and medication usage as they progress through the post-operative recovery period. This was intended for prescribers to track opioid prescription tendencies and patients’ pain perception in their own practice. In essence, it is a way to track pain management efficacy and patient satisfaction. Therefore, we hypothesized that we could create a medication classification system to be used in conjunction with the validated IPA scale to measure physicians’ pain management practice and patient-reported narcotic usage at different post-operative periods.

## 2. Materials and Methods

### 2.1. Patient Demographics

An IRB-approved prospective study for post-operative care was conducted at a Level 1 US Trauma Center over a period of six months. This study enrolled patients at an orthopedic clinic for routine follow-up after operative treatment of fractures, spine pathology, total joint replacement, and other general orthopedic procedures (i.e., rotator cuff repair, hardware removal, etc.) (Supplemental Table S1 for surgical procedures). Informed consent was obtained according to the approved study protocol upon patient presentation to their clinic appointment. Demographic information, such as gender, age, and operative date, was obtained prior to being administered by a provider.

### 2.2. Procedure

All participants were asked to rate their pain level via a paper survey using a validated pain scale: the IPA scale (0 no pain; 1 tolerable pain; 2 intolerable pain). Additionally, they were asked about their daily narcotic consumption. Information was collected on all patients’ recent controlled substance prescriptions, as reported by the Michigan Automated Prescription System (MAPS) narcotics registry, to establish their daily narcotic usage in MMEs [16,17]. Their ICD 10 and CPT codes for the type of surgical intervention were recorded. The patient-reported pain prescriptions were statistically grouped into five alphabetical categories based on the data of the type of opioids most commonly prescribed and medication frequency. The five categories were matched to the daily MMEs from MAPS using statistical tests for classification. The categories were as follows: A (no pain medication), B (over-the-counter medication), C (occasional use of short-acting narcotics), D (consistent/regular use of short-acting narcotics), and E (long-duration or stronger short-acting narcotics).

Patients were further subdivided based on the following post-operative periods: two weeks, six weeks, three months, and six months. The IPA scale associated with each post-operative encounter was incorporated. For example, a patient taking occasional short-acting narcotics and reporting their pain as tolerable with this regimen at two weeks was rated a C1 (C for short-acting occasional use narcotics, and 1 for tolerable pain). Finally, the percentage of patients with no or tolerable pain at each post-operative stage was calculated to provide the physician with an overview of how well they are managing their patients’ pain. The IPA scale combined with the ABCDE MAPS classification and post-operative stage graph was called the Detroit Interventional Pain Assessment, or DIPA. This study was funded by the Rehab Institute of Michigan Foundation (Detroit, MI, USA).

### 2.3. Statistical Analysis

All statistical analyses were conducted using the Statistical Package for the Social Sciences (SPSS Statistics v29; IBM Corp, Armonk, NY, USA) set at a significance level of 0.05. A hierarchical cluster analysis was initially used to identify the number of clusters or narcotic classes (Supplemental Data File S1). This was followed by using a K-means cluster analysis to determine the specifics of the groups (i.e., cluster value ranges) (Supplemental Data File S2). The SPSS generated highest and lowest extreme values for each class; these values were used as cutoff points. To validate ANOVA assumptions, data were assessed with Shapiro–Wilk normality tests. However, due to non-normal distributions, Kruskal–Wallis tests were used to determine the differences between narcotic classes (C, D, and E) (Supplemental Data File S3). For the same reasons, the Mann–Whitney U test was conducted to determine the difference in the percentage of patients on narcotics between two time periods (Supplemental Data File S4). 

### 2.4. Ethical Considerations

Prior to pain assessment interviews, all patients’ consent was verbally recorded, and they were briefed about this study. Patients had the option to opt out at any time. There were minimal risks associated with this study.

## 3. Results

The ICD 10 classification revealed that 502 participants were included in this study. Of those, 225 were male and 277 were female. Patients’ ages ranged from 18 to 87 with an average of 50 ± 16 SD. There were 210 patients after fracture fixation, 138 post-spinal surgery, and 154 total joint replacement patients. Upon further data analyses, the results displayed no significant difference (*p* = 0.163) between pain tolerability and gender (males vs. females) (Supplemental Data File S5).

### 3.1. Scientific Validation of Medication and Grouping

**Figure 1:**
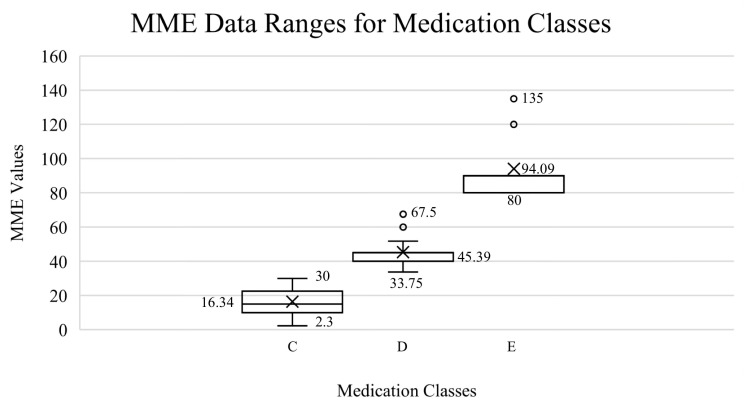
MME ranges for medication classes: C (occasional use of short-acting narcotics), D (consistent use of short-acting narcotics), and E (long-term narcotics/IV use) for post-operative orthopedic intervention. Medication ranges consisted of 2.3–30 MMEs, average 16.34 ± 0.08 (Class C); 33.75–67.5 MMEs, average 45.39 ± 0.22 (Class D); and 80–135 MMEs, average 94.09 ± 17.48 (Class E). “X” represents the averages. “O” represents outliers.

According to the statistical analysis and after daily MME conversions, the narcotic classes corresponded to three MME numerical ranges (Supplemental Table S2). Again, Class A was no pain medication and Class B was over-the-counter medications, both representing 0 MMEs. Narcotic classes C, D, and E significantly differed from each other (*p* < 0.001). Class C patients had a daily MME average of 16.34 ± 0.08 (range 2.3–30) and corresponded to the use of schedule II and IV drugs (codeine–acetaminophen, tramadol, hydrocodone, or oxycodone) on occasion, as needed. Class D included patients who had a daily MME average of 45.39 ± 0.22 (range 33.75–67.5) and represented patients using schedule II drugs (hydrocodone or oxycodone) at regular intervals consistently each day. Occasional use was defined as those who ingested medication as needed or infrequently during post-operative care. Consistent use was defined as patients who consumed narcotics routinely every day during post-operative care. Class E included patients with a daily MME average of 94.09 ± 17.48 (range 80–135) and corresponded to patients using long-acting schedule II narcotics, such as hydromorphone, methadone, morphine, and Oxycontin with breakthrough short-acting medication (Figure 1 Boxplot). There was a strong positive correlation (ρ = 0.721) between the alphabetical classification and MME value at *p* < 0.001, such that a higher class (class E) was more likely to have a larger MME value (Supplemental Data File S1). Similarly, frequency was strongly correlated to MMEs (ρ = 0.661) in the positive direction. Moreover, MMEs and frequency proved to be significant (both *p* < 0.001), discriminating variables when determining the different grouping memberships of narcotics.

### 3.2. Pain Management Score

**Figure 2:**
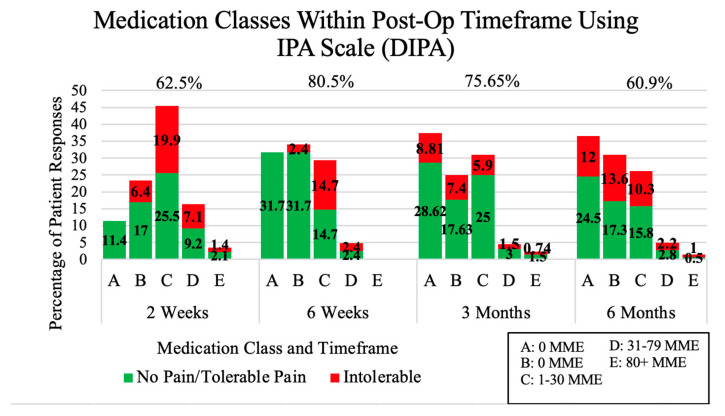
Percentage of total patient responses for their medication class, pain perception percentage, and average MME progression for 2 weeks, 6 weeks, 3 months, and 6 months post-operative. Provider efficiency scores are represented as percentages and show providers are best at managing post-operative pain at 6 weeks and 3 months.

In addition, we wanted to provide a feedback mechanism for providers to evaluate the efficacy of their post-operative pain protocol. Thus, we created a scoring system based on IPA responses which grouped together all treated patients experiencing no pain (0) or tolerable pain (1) and assigned them a value of 1. Patients who rated their pain as intolerable were assigned a value of 0. Next, we added them all up, effectively counting all adequately treated patients, divided the number by the total number of patients, and then multiplied by 100. This represented a percentage of patients who felt they had satisfactory pain management. The percentages were calculated at every post-operative time period. This new IPA pain management score categorized by medication class was represented graphically (Figure 2).

The results of the new IPA pain management score categorized by medication class (DIPA) displayed that the percentage of cases with no pain and tolerable pain was greater than the percentage of cases with intolerable pain in almost every class for each post-operative period. Patients were most satisfied with their pain management at six weeks (80.5%) and three months (75.65%). Lower patient satisfaction scores were represented at two weeks (62.5%) and six months (60.9%). The lower pain scores at two weeks reflected that many patients had run out of their pain medication before their visit at the clinic. Moreover, after reviewing the data, this was attributed to an inadequate number of pain pills prescribed as patients were discharged. New state prescribing rules maintained that a maximum of one week of pain medications can be prescribed at discharge. However, patients were not seen until two weeks post-operative. Additional information displayed on the DIPA graph revealed there was a significant decrease in the percentage of patients on narcotics at two weeks (65.2%) to six months (32.6%) at *p* < 0.001.

## 4. Discussion

Pain is a complex patient experience that is frequently assessed and used as an objective piece of data along with other vital signs [18]. As healthcare and pain management guidelines continue to change, surgeons are still looking for better ways to manage their patients’ post-operative pain [19].

In order to study any metric, there must be a way to measure it. In a previous work, the patient-preferred IPA scale has been validated and exhibited a statistically significant correlation with the NRS [9]. Next, we developed the DIPA scale, which is one of the first pain scales to incorporate both pain tolerance and medication use. We found that most post-operative patients in our clinics were prescribed narcotics during the immediate post-operative period, which necessitates our providers to rely on MAPS for daily MME values. Through the addition of MME-assigned medication classes to the IPA scale, the new “DIPA” scale can provide a greater understanding of a patient’s or a group of patients’ pain perceptions and medication use. In this study, medication usage ranged from no medications (A 0 MMEs) to long-acting narcotics with breakthrough medications (E 80+ MMEs). MME values were no longer arbitrary, but rather translated into simple understandable categories, such as C = occasional narcotic usage, D = consistent daily narcotic usage, or E = long-acting narcotics with breakthrough short-acting medications. Here, the satisfaction scores fluctuated from 60.9% to 80.5% for the medications that were being used at the post-operative time category specific to the group. This can potentially help evaluate the success of orthopedic pain management strategies.

Previous efforts to create a more meaningful use of MME values have been published. Fulton-Kehoe et al. created a medication classification system with meaningful definitions in conjunction with MME daily values using six categories [20]. Within those categories, they used ≥50 MMEs (chronic high-dose opioid therapy) and ≥90 MMEs (risk for overdose and respiratory depression) as reference guides. While this scale is a step in the right direction in impacting healthcare practices, it still fails to associate specific narcotics with MME values.

The statistical analyses demonstrate that the DIPA scale is reliable, valid, and applicable to our orthopedic clinic population. Additionally, any orthopedic practitioner can implement this scale in their clinic by asking their patients the following three questions: (1) Do you have no pain, tolerable pain, or intolerable pain? (2) What pain medication are you taking? (3) Do you take it occasionally as needed or consistently on a daily basis? From there, providers can group patients according to the DIPA scale. For example, if a patient reports their pain as tolerable, taking Norco 5 mg occasionally (as needed), then they are rated as a C1. Without having to refer to MAPS, the provider knows the patient’s daily MME value lies between 1–30 MMEs. Therefore, physicians can use this as a reference guide to longitudinally track their pain management efficacy and prescribe tendencies across post-operative periods.

Limitations of this study include the cohort of patients. The surveys were conducted on all adult patients. Therefore, the IPA scale should be tested against a pediatric group to assess its validity in a vulnerable population. We did not assess for the use of other pain modalities, such as marijuana or illicit drugs, which would provide a broader understanding of pain perception. There were patients who used narcotics prior to surgery and may be using them for other diagnoses. This could include patients with chronic diseases that tend to increase the amount of pain experienced by patients. This study did not take this into account, and a review of intake histories and medication reconciliation data for those people could be performed in a future study. This is where fracture patients may differ from degenerative diseases, such as arthritis and back pain. We did not question why patients were taking pain medications at six months, which may solve these questions and could discover risk factors for continued use. Another limitation is the applicability of the DIPA scale. This study proves that the DIPA scale is only applicable to orthopedic providers. While its intended use is for it to eventually be applied to all cohorts of patients, more studies need to be performed within different patient populations for it to be validated. Lastly, this is a single-center study. Including a more diverse patient participant pool by surveying patients with the DIPA scale at other hospitals and clinics is a valuable future effort.

The purpose of including a wide range of orthopedic patients was to show that the DIPA scale can be broadly applied across all orthopedic specialties. However, the next step should be to apply the DIPA scale to a specific population of orthopedic patients, such as those receiving fracture care, to determine providers’ prescription tendencies and patient pain satisfaction.

Overall, the simplistic versatility and multidimensionality of the DIPA scale promote its usage in different pain management scenarios as well as its application as a longitudinal assessment tool. Once a problem can be measured, as in the case of patient pain satisfaction and narcotic usage, physicians can then study the problem more effectively in a way that produces better outcomes.

## 5. Conclusions

The DIPA scale and assessment tool demonstrates the ability to evaluate pain perception, medication usage, and can track pain management efficacy. Providers were able to visualize their post-operative pain management progression at each designated clinic visit with corresponding alphabetical daily MME categories. In this study, results suggest that surgeons were not effective at managing patients’ pain at two weeks post-operative, which was attributed to an inadequate number of pain pills being prescribed upon discharge. However, in subsequent clinic visits at six weeks and three months, providers’ pain management practice improved, and was evidenced by a significant decrease in the percentage of patients on narcotics and corresponding higher efficiency scores. Overall, the DIPA graph signaled that better pain management interventions are necessitated in periods with lower efficiency scores.

## Data Availability

The data, statistical results, and supplemental materials are available upon reasonable request from the authors of this study. The data are stored as de-identified participant data in Excel files. Please email rahvaidya2012@gmail.com for data requests.

## Supplementary Materials

The following supporting information can be downloaded at: https://www.mdpi.com/article/10.3390/medicina59111976/s1, Supplemental Data File S1: Hierarchical cluster analysis of the number of narcotic groups. Supplemental Data File S2: K-means cluster analysis determining the specifics of narcotic groups (value ranges, mean, etc.). Supplemental Data File S3: Kruskal-Wallis test displaying the differences between narcotic classes C, D, and E. Supplemental Data File S4: Mann-Whitney U test displaying the significant difference between percentage of pa-tients on narcotics at 2 weeks and 6 months. Supplemental Data File S5: Displays the difference between pain tolerability and gender (males vs females). No sig-nificant difference was observed. Supplemental Table S1: Orthopedic surgical procedures for all patients. Procedures consisted of patients undergo-ing fracture, joint, and spinal intervention. Supplemental Table S2: MME conversations for all narcotic groups (C, D, and E).
