# Supplementary material for: Detroit Interventional Pain Assessment Scale: A Pain Score and Method for Measuring and Evaluating Post-Operative Pain Management—A Prospective Study
Source: Medicina (Kaunas). 2023 Nov 9;59(11):1976. doi: 10.3390/medicina59111976 (PMC10673537; doi:10.3390/medicina59111976)
Supplement: Supplementary file 1 [file medicina-2627174-supplementary.pdf › medicina-2627174-supplementary.pdf]

Narcotic	Frequency	MME	Time	CLU3_1	QCL_1	QCL_2	QCL_3	QCL_4	QCL_5
Tylenol 3 1	2	51.80	3	3	2	2.86627	2	2	6.27053
Oxycodone	4	60.00	1	1	2	5.59938	2	2	14.42498
Percocet 1	4	60.00	1	1	2	5.59938	2	2	14.42498
Oxycodone	3	45.00	1	3	2	9.49515	2	2	0.60972
Oxycodone	3	45.00	1	3	2	9.49515	2	2	0.60972
Oxycodone	3	45.00	1	3	2	9.49515	2	2	0.60972
Percocet 1	3	45.00	3	3	2	9.49515	2	2	0.60972
Percocet 1	3	45.00	1	3	2	9.49515	2	2	0.60972
Percocet 1	3	45.00	1	3	2	9.49515	2	2	0.60972
Percocet 1	3	45.00	1	3	2	9.49515	2	2	0.60972
Percocet 1	3	45.00	1	3	2	9.49515	2	2	0.60972
Percocet 1	3	45.00	1	3	2	9.49515	2	2	0.60972
Percocet 1	3	45.00	4	3	2	9.49515	2	2	0.60972
Percocet 1	3	45.00	4	3	2	9.49515	2	2	0.60972
Oxycodone	2	45.00	2	3	2	9.54511	2	2	1.17122
Oxycodone	2	45.00	1	3	2	9.54511	2	2	1.17122
Percocet 1	2	45.00	3	3	2	9.54511	2	2	1.17122
Percocet 1	2	45.00	4	3	2	9.54511	2	2	1.17122
Percocet 1	2	45.00	4	3	2	9.54511	2	2	1.17122
Oxycodone	4	45.00	1	3	2	9.55022	2	2	1.17122
Percocet 7	4	45.00	1	3	2	9.55022	2	2	1.17122
Percocet 1	1	45.00	4	3	2	9.69847	2	2	2.09088
Oxycodone	3	67.50	1	1	2	13.00490	2	2	21.89028
Oxycodone	3	67.50	3	1	2	13.00490	2	2	21.89028
Oxycodone	2	67.50	4	1	2	13.04142	2	2	21.91311
Norco 10m	4	40.00	1	3	2	14.53127	2	2	5.69816
Norco 10m	4	40.00	3	3	2	14.53127	2	2	5.69816
Norco 10m	4	40.00	1	3	2	14.53127	2	2	5.69816
Norco 10m	4	40.00	1	3	2	14.53127	2	2	5.69816
Norco 10m	4	40.00	1	3	2	14.53127	2	2	5.69816
Norco 10m	4	40.00	1	3	2	14.53127	2	2	5.69816
Norco 7.5	4	37.50	1	3	2	17.02597	2	2	8.17114
Norco 7.5	2	15.00	1	2	3	1.83234	3	1	1.41969
Norco 7.5	2	15.00	3	2	3	1.83234	3	1	1.41969
Norco 7.5	2	15.00	1	2	3	1.83234	3	1	1.41969
Norco 7.5	2	15.00	3	2	3	1.83234	3	1	1.41969
Norco 7.5	2	15.00	1	2	3	1.83234	3	1	1.41969
Norco 7.5	2	15.00	3	2	3	1.83234	3	1	1.41969
Norco 7.5	2	15.00	3	2	3	1.83234	3	1	1.41969
Norco 7.5	2	15.00	1	2	3	1.83234	3	1	1.41969
Norco 7.5	2	15.00	4	2	3	1.83234	3	1	1.41969
Norco 7.5	2	15.00	3	2	3	1.83234	3	1	1.41969
Norco 7.5	2	15.00	2	2	3	1.83234	3	1	1.41969
Oxycodone	2	15.00	3	2	3	1.83234	3	1	1.41969
Oxycodone	2	15.00	1	2	3	1.83234	3	1	1.41969
Percocet 7	2	15.00	1	2	3	1.83234	3	1	1.41969
Percocet 1	1	15.00	1	2	3	2.05874	3	1	1.69387
Percocet 1	1	15.00	1	2	3	2.05874	3	1	1.69387
Percocet 5	1	15.00	3	2	3	2.05874	3	1	1.69387
Tramadol 5	1	15.00	4	2	3	2.05874	3	1	1.69387
Norco 5 m	3	15.00	3	2	3	2.11577	3	1	1.77816

Norco 5 m	3	15.00	1	2	3	2.11577	3	1	1.77816
Norco 5 m	3	15.00	1	2	3	2.11577	3	1	1.77816
Norco 5 m	3	15.00	1	2	3	2.11577	3	1	1.77816
Norco 5 m	3	15.00	3	2	3	2.11577	3	1	1.77816
Norco 5 m	3	15.00	3	2	3	2.11577	3	1	1.77816
Norco 5 m	3	15.00	1	2	3	2.11577	3	1	1.77816
Norco 5 m	3	15.00	1	2	3	2.11577	3	1	1.77816
Norco 5 m	3	15.00	4	2	3	2.11577	3	1	1.77816
Norco 10m	2	20.00	1	2	3	3.16919	3	1	3.58294
Norco 10m	2	20.00	1	2	3	3.16919	3	1	3.58294
Norco 10m	2	20.00	1	2	3	3.16919	3	1	3.58294
Norco 10m	2	20.00	3	2	3	3.16919	3	1	3.58294
Norco 10m	2	20.00	3	2	3	3.16919	3	1	3.58294
Norco 10m	2	20.00	3	2	3	3.16919	3	1	3.58294
Norco 10m	2	20.00	2	2	3	3.16919	3	1	3.58294
Norco 10m	2	20.00	3	2	3	3.16919	3	1	3.58294
Norco 10m	2	20.00	3	2	3	3.16919	3	1	3.58294
Norco 10m	2	20.00	3	2	3	3.16919	3	1	3.58294
Norco 10m	2	20.00	3	2	3	3.16919	3	1	3.58294
Norco 10m	2	20.00	2	2	3	3.16919	3	1	3.58294
Norco 10m	2	20.00	1	2	3	3.16919	3	1	3.58294
Norco 10m	2	20.00	1	2	3	3.16919	3	1	3.58294
Norco 10m	2	20.00	2	2	3	3.16919	3	1	3.58294
Norco 10m	2	20.00	2	2	3	3.16919	3	1	3.58294
Norco 10m	2	20.00	1	2	3	3.16919	3	1	3.58294
Norco 5 m	2	20.00	3	2	3	3.16919	3	1	3.58294
Percocet 1	2	20.00	4	2	3	3.16919	3	1	3.58294
Tramadol 5	2	20.00	4	2	3	3.16919	3	1	3.58294
Norco 5 m	4	20.00	1	2	3	3.77913	3	1	4.13886
Norco 5 m	4	20.00	1	2	3	3.77913	3	1	4.13886
Oxycodone	1	11.30	4	2	3	5.61075	3	1	5.20105
Oxycodone	1	11.30	4	2	3	5.61075	3	1	5.20105
Percocet 7	2	22.50	1	2	3	5.66894	3	1	6.08264
Percocet 1	1	22.50	4	2	3	5.74612	3	1	6.15241
Percocet 1	1	22.50	4	2	3	5.74612	3	1	6.15241
Norco 7.5	3	22.50	1	2	3	5.76680	3	1	6.17615
Norco 7.5	3	22.50	1	2	3	5.76680	3	1	6.17615
Norco 7.5	3	22.50	3	2	3	5.76680	3	1	6.17615
Norco 7.5	3	22.50	1	2	3	5.76680	3	1	6.17615
Norco 7.5	3	22.50	3	2	3	5.76680	3	1	6.17615
Norco 7.5	3	22.50	1	2	3	5.76680	3	1	6.17615
Norco 7.5	3	22.50	1	2	3	5.76680	3	1	6.17615
Oxycodone	3	22.50	1	2	3	5.76680	3	1	6.17615
Percocet 5	3	22.50	1	2	3	5.76680	3	1	6.17615
Norco 7.5	2	22.77	4	2	3	5.93893	3	1	6.35262
Norco 10m	2	10.00	3	2	3	6.83163	3	1	6.41822
Norco 5 m	2	10.00	1	2	3	6.83163	3	1	6.41822
Norco 5 m	2	10.00	4	2	3	6.83163	3	1	6.41822
Norco 5 m	2	10.00	1	2	3	6.83163	3	1	6.41822
Norco 5 m	2	10.00	3	2	3	6.83163	3	1	6.41822
Norco 5 m	2	10.00	1	2	3	6.83163	3	1	6.41822
Norco 5 m	2	10.00	2	2	3	6.83163	3	1	6.41822

Norco 5 m	2	10.00	1	2	3	6.83163	3	1	6.41822
Norco 5 m	2	10.00	4	2	3	6.83163	3	1	6.41822
Norco 7.5	2	10.00	1	2	3	6.83163	3	1	6.41822
Tramadol 5	2	10.00	3	2	3	6.83163	3	1	6.41822
Tramadol 5	2	10.00	1	2	3	6.83163	3	1	6.41822
Tramadol 5	2	10.00	4	2	3	6.83163	3	1	6.41822
Tramadol 5	2	10.00	4	2	3	6.83163	3	1	6.41822
Norco 10m	1	10.00	4	2	3	6.89580	3	1	6.48438
Norco 10m	1	10.00	4	2	3	6.89580	3	1	6.48438
Norco 10m	1	10.00	4	2	3	6.89580	3	1	6.48438
Norco 10m	1	10.00	1	2	3	6.89580	3	1	6.48438
Norco 10m	1	10.00	3	2	3	6.89580	3	1	6.48438
Norco 10m	1	10.00	4	2	3	6.89580	3	1	6.48438
Norco 10m	1	10.00	3	2	3	6.89580	3	1	6.48438
Norco 10m	1	10.00	3	2	3	6.89580	3	1	6.48438
Norco 10m	1	10.00	3	2	3	6.89580	3	1	6.48438
Norco 10m	1	10.00	4	2	3	6.89580	3	1	6.48438
Norco 10m	1	10.00	4	2	3	6.89580	3	1	6.48438
Norco 10m	1	10.00	4	2	3	6.89580	3	1	6.48438
Tramadol 5	1	10.00	2	2	3	6.89580	3	1	6.48438
Tylenol 4 6	1	9.00	4	2	3	7.88764	3	1	7.47548
Percocet 7	2	7.50	4	2	3	9.33156	3	1	8.91811
Norco 7.5	1	7.50	4	2	3	9.37864	3	1	8.96584
Norco 7.5	1	7.50	4	2	3	9.37864	3	1	8.96584
Norco 7.5	1	7.50	3	2	3	9.37864	3	1	8.96584
Norco 7.5	1	7.50	3	2	3	9.37864	3	1	8.96584
Norco 7.5	1	7.50	3	2	3	9.37864	3	1	8.96584
Norco 7.5	1	7.50	2	2	3	9.37864	3	1	8.96584
Norco 7.5	1	7.50	2	2	3	9.37864	3	1	8.96584
Norco 7.5	1	7.50	2	2	3	9.37864	3	1	8.96584
Norco 7.5	1	7.50	3	2	3	9.37864	3	1	8.96584
Norco 7.5	1	7.50	4	2	3	9.37864	3	1	8.96584
Norco 7.5	1	7.50	4	2	3	9.37864	3	1	8.96584
Norco 7.5	1	7.50	4	2	3	9.37864	3	1	8.96584
Percocet 5	1	7.50	1	2	3	9.37864	3	1	8.96584
Percocet 5	1	7.50	1	2	3	9.37864	3	1	8.96584
Tylenol 3 1	3	6.80	2	2	3	10.08717	3	1	9.67749
Norco 7.5	1	28.13	2	3	3	11.33771	3	1	11.74881
Norco 10m	3	28.13	1	3	3	11.34820	3	1	11.76126
Norco 7.5	3	28.13	4	3	3	11.34820	3	1	11.76126
Norco 7.5	3	28.13	3	3	3	11.34820	3	1	11.76126
Percocet 1	3	28.13	1	3	3	11.34820	3	1	11.76126
Norco 5 m	1	5.00	4	2	3	11.86869	3	1	11.45536
Norco 5 m	1	5.00	3	2	3	11.86869	3	1	11.45536
Norco 5 m	1	5.00	2	2	3	11.86869	3	1	11.45536
Norco 5 m	1	5.00	3	2	3	11.86869	3	1	11.45536
Norco 5 m	1	5.00	4	2	3	11.86869	3	1	11.45536
Norco 5 m	1	5.00	1	2	3	11.86869	3	1	11.45536
Norco 5 m	1	5.00	1	2	3	11.86869	3	1	11.45536
Norco 5 m	1	5.00	1	2	3	11.86869	3	1	11.45536
Norco 5 m	1	5.00	2	2	3	11.86869	3	1	11.45536
Tramadol 5	1	5.00	1	2	3	11.86869	3	1	11.45536

Tramadol 5	1	5.00	4	2	3	11.86869	3	1	11.45536
Tramadol 5	1	5.00	2	2	3	11.86869	3	1	11.45536
Tramadol 5	1	5.00	2	2	3	11.86869	3	1	11.45536
Tramadol 5	1	5.00	4	2	3	11.86869	3	1	11.45536
Tramadol 5	1	5.00	4	2	3	11.86869	3	1	11.45536
Tylenol 4 1	2	4.50	4	2	3	12.33151	3	1	11.91803
Tylenol 4 1	1	4.50	4	2	3	12.36718	3	1	11.95379
Oxycodone	2	30.00	1	3	3	13.16877	3	1	13.58239
Oxycodone	2	30.00	1	3	3	13.16877	3	1	13.58239
Percocet 1	2	30.00	1	3	3	13.16877	3	1	13.58239
Percocet 1	2	30.00	1	3	3	13.16877	3	1	13.58239
Percocet 1	2	30.00	1	3	3	13.16877	3	1	13.58239
Percocet 1	2	30.00	1	3	3	13.16877	3	1	13.58239
Percocet 1	2	30.00	4	3	3	13.16877	3	1	13.58239
Percocet 1	2	30.00	4	3	3	13.16877	3	1	13.58239
Percocet 1	2	30.00	4	3	3	13.16877	3	1	13.58239
Percocet 1	2	30.00	1	3	3	13.16877	3	1	13.58239
Norco 10m	1	30.00	4	3	3	13.20217	3	1	13.61378
Norco 10m	3	30.00	1	3	3	13.21119	3	1	13.62453
Norco 10m	3	30.00	1	3	3	13.21119	3	1	13.62453
Norco 10m	3	30.00	1	3	3	13.21119	3	1	13.62453
Norco 10m	3	30.00	1	3	3	13.21119	3	1	13.62453
Norco 10m	3	30.00	1	3	3	13.21119	3	1	13.62453
Norco 10m	3	30.00	2	3	3	13.21119	3	1	13.62453
Norco 10m	3	30.00	3	3	3	13.21119	3	1	13.62453
Norco 10m	3	30.00	4	3	3	13.21119	3	1	13.62453
Norco 10m	3	30.00	2	3	3	13.21119	3	1	13.62453
Norco 10m	3	30.00	4	3	3	13.21119	3	1	13.62453
Norco 10m	3	30.00	4	3	3	13.21119	3	1	13.62453
Norco 10m	3	30.00	3	3	3	13.21119	3	1	13.62453
Norco 7.5	3	30.00	4	3	3	13.21119	3	1	13.62453
Norco 7.5	3	30.00	3	3	3	13.21119	3	1	13.62453
Norco 7.5	3	30.00	4	3	3	13.21119	3	1	13.62453
Norco 7.5	3	30.00	3	3	3	13.21119	3	1	13.62453
Percocet 1	3	30.00	4	3	3	13.21119	3	1	13.62453
Percocet 5	3	30.00	3	3	3	13.21119	3	1	13.62453
Percocet 5	3	30.00	3	3	3	13.21119	3	1	13.62453
Tylenol 3 1	1	2.30	4	2	3	14.56177	3	1	14.14820
Tylenol 3 1	1	2.30	4	2	3	14.56177	3	1	14.14820
Tylenol 3 1	1	2.30	3	2	3	14.56177	3	1	14.14820
Tylenol 3 1	1	2.30	4	2	3	14.56177	3	1	14.14820
Percocet 1	1	33.75	3	3	3	16.94475	3	2	12.02718
Oxycodone	3	33.80	1	3	3	17.00168	3	2	11.80972
Oxycodone	3	33.80	1	3	3	17.00168	3	2	11.80972
Percocet 7	3	33.80	1	3	3	17.00168	3	2	11.80972

Supplemental Data File S2

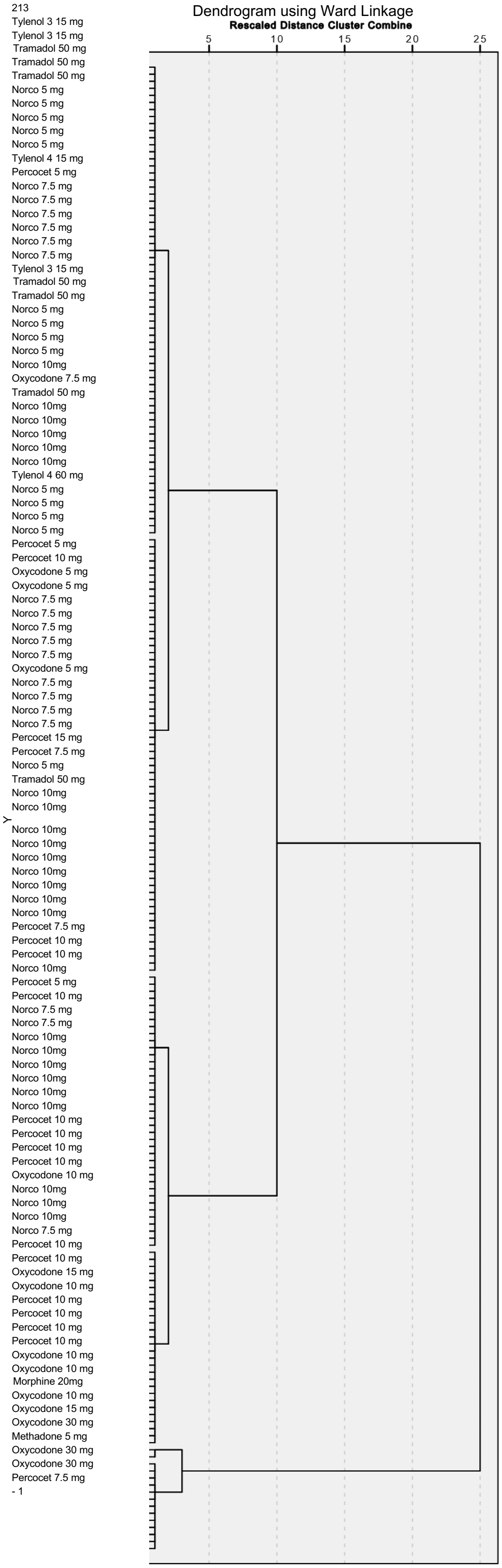

Quick Cluster

Initial Cluster Centers

	Cluster	
	C	D
MME	2.30	67.50
Frequency	1	3

Iteration History[a]

	Change in Cluster Centers	
Iteration	C	D
1	14.562	20.413
2	.414	1.479
3	.000	.000

a. Convergence achieved due to no or small change in cluster centers. The maximum absolute coordinate change for any center is .000. The current iteration is 3. The minimum distance between initial centers is 65.231.

Final Cluster Centers

	Cluster	
	C	D
MME	16.42	45.61
Frequency	2	3

ANOVA

	Cluster		Erro			
	Mean Square	df	Mean Square	df	F	Sig.
MME	25156.000	1	77.240	198	325.687	<.001
Frequency	33.998	1	.672	198	50.567	<.001

The F tests should be used only for descriptive purposes because the clusters have been chosen to maximize the differences among cases in different clusters. The observed significance levels are not corrected for this and thus cannot be interpreted as tests of the hypothesis that the cluster means are equal.

Number of Cases in each Cluster

Cluster	C	164.000
	D	36.000

Valid	200.000
Missing	.000

Tests of Normality

	Class	Kolmogorov-Smirnov[a] Statistic	df	Sig.	Shapiro-Wilk Statistic	df	Sig.
MME	C	.164	162	<.001	.906	162	<.001
	D	.356	37	<.001	.790	37	<.001
	E	.428	9	<.001	.615	9	<.001

a. Lilliefors Significance Correction

MME

Histograms

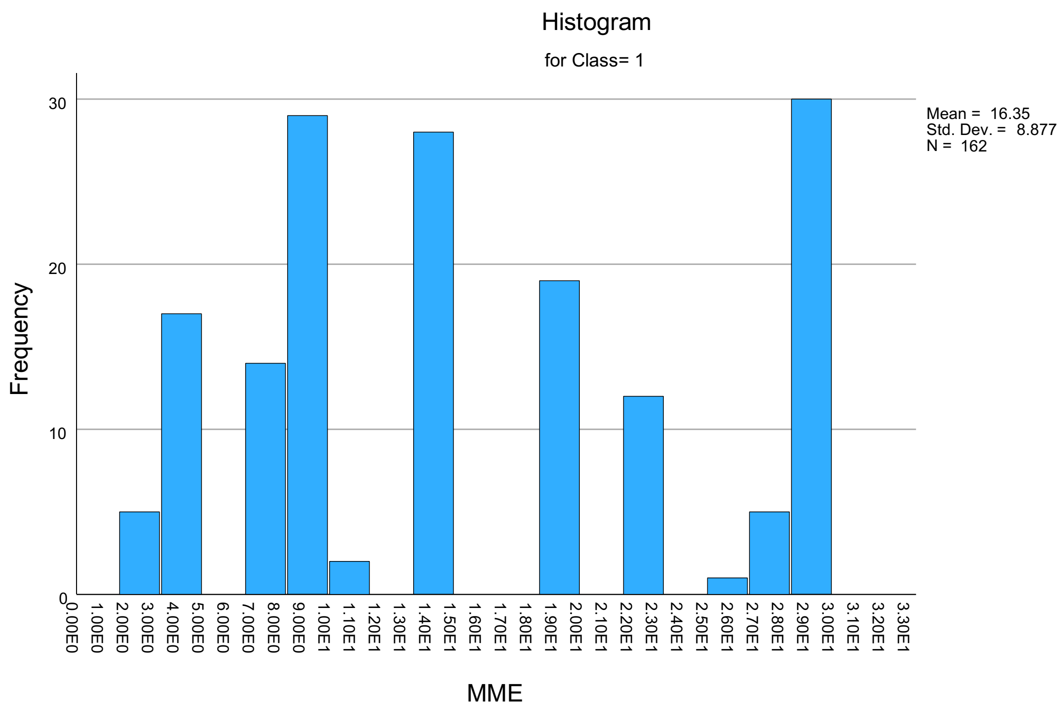

Kruskal-Wallis Test

Ranks

	Class	N	Mean Rank
**MME**	**C**	**162**	**81.50**

	D	37	181.00
	E	9	204.00
	Total	208	

Test Statistics[a,b]

	MME
Kruskal-Wallis H	109.140
df	2
Asymp. Sig.	<.001

a. Kruskal Wallis Test

b. Grouping Variable: Class

NPar Tests

Mann-Whitney Test

Ranks

	Class	N	Mean Rank	Sum of Ranks
MME	C	162	81.50	13203.00
	D	37	181.00	6697.00
	Total	199		

Test Statistics[a]

	MME
Mann-Whitney U	.000
Wilcoxon W	13203.000
Z	-9.538
Asymp. Sig. (2-tailed)	<.001

a. Grouping Variable: Class

NPar Tests

Mann-Whitney Test

Ranks

	Class	N	Mean Rank	Sum of Ranks
MME	D	37	19.00	703.00
	E	9	42.00	378.00

	Total	46		

Test Statistics[a]

	MME
Mann-Whitney U	.000
Wilcoxon W	703.000
Z	-4.792
Asymp. Sig. (2-tailed)	<.001
Exact Sig. [2*(1-tailed Sig.)]	<.001[b]

a. Grouping Variable: Class

b. Not corrected for ties.

NPar Tests

Mann-Whitney Test

Ranks

	Class	N	Mean Rank	Sum of Ranks
MME	C	162	81.50	13203.00
	E	9	167.00	1503.00
	Total	171		

Test Statistics[a]

	MME
Mann-Whitney U	.000
Wilcoxon W	13203.000
Z	-5.086
Asymp. Sig. (2-tailed)	<.001

a. Grouping Variable: Class

Tests of Normality

	Frequency	Kolmogorov-Smirnov[a] Statistic	df	Sig.	Shapiro-Wilk Statistic	df	Sig.
MME	1	.301	55	<.001	.541	55	<.001
	2	.288	74	<.001	.697	74	<.001
	3	.219	66	<.001	.906	66	<.001
	4	.274	13	.009	.841	13	.022
Class	1	.535	55	<.001	.117	55	<.001
	2	.479	74	<.001	.507	74	<.001
	3	.456	66	<.001	.557	66	<.001
	4	.408	13	<.001	.675	13	<.001

a. Lilliefors Significance Correction

MME

Normal Q-Q Plots

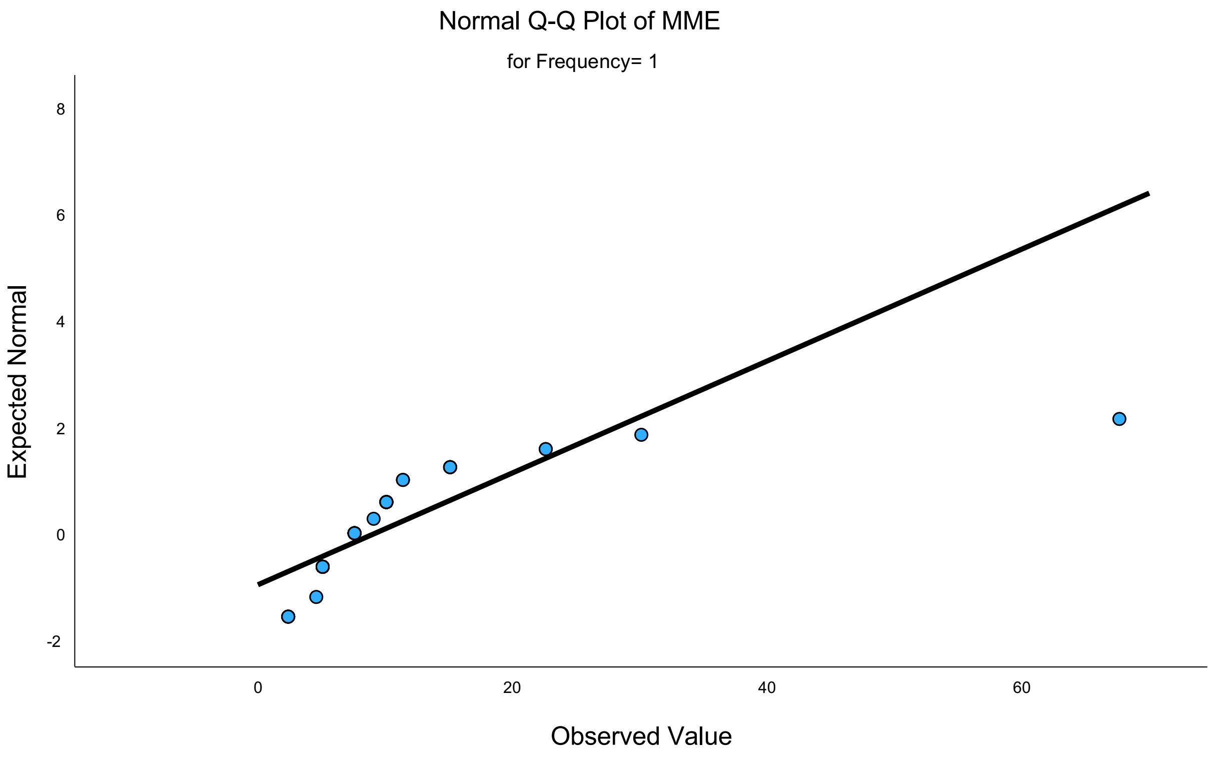

Kruskal-Wallis Test

Ranks

	Frequency	N	Mean Rank
MME	1	55	41.45
	2	74	110.68
	3	66	136.80
	4	13	172.08
	Total	208	
Class	1	55	83.80
	2	74	104.80
	3	66	109.00
	4	13	167.50
	Total	208	

Test Statistics[a,b]

	MME	Class
Kruskal-Wallis H	97.546	41.097
df	3	3
Asymp. Sig.	<.001	<.001

a. Kruskal Wallis Test

b. Grouping Variable: Frequency

Nonparametric Correlations

Correlations

			Frequency	MME	Class
Spearman's rho	Frequency	Correlation Coefficient	1.000	.661**	.360**
		Sig. (1-tailed)	.	<.001	<.001
		N	208	208	208
	MME	Correlation Coefficient	.661**	1.000	.721**
		Sig. (1-tailed)	<.001	.	<.001
		N	208	208	208
	Class	Correlation Coefficient	.360**	.721**	1.000
		Sig. (1-tailed)	<.001	<.001	.

N	208	208	208

**. Correlation is significant at the 0.01 level (1-tailed).

Case Processing Summary

	time	Cases					
		Valid		Missing		Total	
		N	Percent	N	Percent	N	Percent
Percentage Patients on narcotics	2 weeks	141	100.0%	0	0.0%	141	100.0%
	6 weeks	43	100.0%	0	0.0%	43	100.0%
	3 months	136	100.0%	0	0.0%	136	100.0%
	6 months	182	100.0%	0	0.0%	182	100.0%

Descriptives

	time			Statistic	Std. Error
Percentage Patients on narcotics	2 weeks	Mean		65.2482	4.02447
		95% Confidence Interval for Mean	Lower Bound	57.2916	
			Upper Bound	73.2048	
		5% Trimmed Mean		66.9425	
		Median		100.0000	
		Variance		2283.688	
		Std. Deviation		47.78795	
		Minimum		.00	
		Maximum		100.00	
		Range		100.00	
		Interquartile Range		100.00	
		Skewness		-.647	.204
		Kurtosis		-1.604	.406
	6 weeks	Mean		37.2093	7.45845
		95% Confidence Interval for Mean	Lower Bound	22.1575	
			Upper Bound	52.2611	
		5% Trimmed Mean		35.7881	
		Median		.0000	
		Variance		2392.027	
		Std. Deviation		48.90835	
		Minimum		.00	
		Maximum		100.00	
		Range		100.00	
		Interquartile Range		100.00	
		Skewness		.549	.361
		Kurtosis		-1.784	.709
	3 months	Mean		37.5000	4.16667
		95% Confidence Interval for Mean	Lower Bound	29.2596	
			Upper Bound	45.7404	
		5% Trimmed Mean		36.1111	

				Statistic	Std. Error
		Median		.0000	
		Variance		2361.111	
		Std. Deviation		48.59127	
		Minimum		.00	
		Maximum		100.00	
		Range		100.00	
		Interquartile Range		100.00	
		Skewness		.522	.208
		Kurtosis		-1.753	.413
	6 months	Mean		31.8681	3.46349
		95% Confidence Interval for Mean	Lower Bound	25.0341	
			Upper Bound	38.7021	
		5% Trimmed Mean		29.8535	
		Median		.0000	
		Variance		2183.231	
		Std. Deviation		46.72506	
		Minimum		.00	
		Maximum		100.00	
		Range		100.00	
		Interquartile Range		100.00	
		Skewness		.785	.180
		Kurtosis		-1.400	.358

Tests of Normality

		Kolmogorov-Smirnov[a]			Shapiro-Wilk		
	time	Statistic	df	Sig.	Statistic	df	Sig.
Percentage Patients on narcotics	2 weeks	.419	141	<.001	.602	141	<.001
	6 weeks	.405	43	<.001	.613	43	<.001
	3 months	.405	136	<.001	.613	136	<.001
	6 months	.434	182	<.001	.587	182	<.001

a. Lilliefors Significance Correction

Test of Homogeneity of Variance

		Levene Statistic	df1	df2	Sig.
Percentage Patients on narcotics	Based on Mean	1.624	3	498	.183
	Based on Median	.409	3	498	.746
	Based on Median and with adjusted df	.409	3	497.401	.746
	Based on trimmed mean	1.624	3	498	.183

NPar Tests

Mann-Whitney Test

Ranks

	time	N	Mean Rank	Sum of Ranks
Percentage Patients on narcotics	2 weeks	141	192.38	27125.00
	6 months	182	138.47	25201.00
	Total	323		

Test Statistics[a]

	Percentage_narcotics
Mann-Whitney U	8548.000
Wilcoxon W	25201.000
Z	-5.957
Asymp. Sig. (2-tailed)	<.001

a. Grouping Variable: time

Gender

Case Processing Summary

		Cases					
		Valid		Missing		Total	
	Gender	N	Percent	N	Percent	N	Percent
Tolerable	Male	223	100.0%	0	0.0%	223	100.0%
	Female	279	100.0%	0	0.0%	279	100.0%

Descriptives

	Gender			Statistic	Std. Error
Tolerable	Male	Mean		70.4036	3.06366
		95% Confidence Interval for Mean	Lower Bound	64.3660	
			Upper Bound	76.4412	
		5% Trimmed Mean		72.6707	
		Median		100.0000	
		Variance		2093.080	
		Std. Deviation		45.75019	
		Minimum		.00	
		Maximum		100.00	
		Range		100.00	
		Interquartile Range		100.00	
		Skewness		-.900	.163
		Kurtosis		-1.201	.324
	Female	Mean		64.5161	2.86964
		95% Confidence Interval for Mean	Lower Bound	58.8671	
			Upper Bound	70.1651	
		5% Trimmed Mean		66.1290	
		Median		100.0000	
		Variance		2297.517	
		Std. Deviation		47.93242	
		Minimum		.00	
		Maximum		100.00	
		Range		100.00	
		Interquartile Range		100.00	
		Skewness		-.610	.146
		Kurtosis		-1.640	.291

Tests of Normality

	Kolmogorov-Smirnov[a]			Shapiro-Wilk		
Gender	Statistic	df	Sig.	Statistic	df	Sig.

Tolerable	Male	.445	223	<.001	.573	223	<.001
	Female	.416	279	<.001	.605	279	<.001

a. Lilliefors Significance Correction

Test of Homogeneity of Variance

		Levene Statistic	df1	df2	Sig.
Tolerable	Based on Mean	7.985	1	500	.005
	Based on Median	1.947	1	500	.164
	Based on Median and with adjusted df	1.947	1	498.943	.164
	Based on trimmed mean	7.985	1	500	.005

NPar Tests

Mann-Whitney Test

Ranks

	Gender	N	Mean Rank	Sum of Ranks
Tolerable	Male	223	259.71	57916.00
	Female	279	244.94	68337.00
	Total	502		

Test Statistics[a]

	Tolerable
Mann-Whitney U	29277.000
Wilcoxon W	68337.000
Z	-1.394
Asymp. Sig. (2-tailed)	.163

a. Grouping Variable: Gender

Supplemental Table S1

Sex	IPA scale	Surgicul Procedure
M	0	ORIF right Ankle
M	0	ORIF left Ankle
M	0	IM Rodding Femur
M	0	ORIF left Ankle
F	0	ORIF left Ankle
F	0	Closed IM Rodding right Tibia
F	0	ORIF left Ankle
F	0	ORIF Right Radius
M	0	Arthrodesis left ankle
M	0	ORIF right Talus
M	0	ORIF right Ankle
M	0	ORIF Left Foot
F	0	ORIF right Ankle
F	0	ORIF Radius/Ulna (Left, Wrist)
F	0	ORIF right Ankle
M	0	ORIF right Ankle
F	0	ORIF right Ankle
M	0	ORIF left Tibia
M	0	ORIF left Tibia
M	0	ORIF left Tibial Plateau
M	0	ORIF right Tibial Plateau
F	0	ORIF radius/ulna right wrist
F	0	ORIF fibula
M	0	ORIF Right Clavicle
M	0	ORIF left Calcaneus
M	1	Closed IM rodding right Tibia
M	1	Arthroscopy Knee Surgery (Right, Knee) / ORIF right foot. Ex fix tibia plateau
M	1	Arthroscopy Knee Surgery (Bilateral, Knee)
F	1	Remove hardware left ankle; ORIF left ankle
M	1	ORIF right foot
F	1	ORIF right Tibia
M	1	Remove Hardware Lower Limb (Right, Ankle) / ORIF right ankle
F	1	ORIF left Ankle
F	1	Closed IM rodding right Tibia
F	1	Closed IM Rodding left Femur
F	1	Closed IM Rodding left Femur
M	1	ORIF distal right Humerus Distal
M	1	ORIF right Tibia
F	1	ORIF left Acetabelum
M	1	Closed IM rodding left femur
M	1	Closed IM rodding left femur
M	1	Closed IM rodding right femur
F	1	ORIF left Ankle
M	1	ORIF right Talus
M	1	ORIF left wrist
F	1	ORIF left Ankle
F	1	ORIF right Radius
M	1	ORIF right Foot
M	1	Closed IM rodding right femur
F	1	ORIF right Foot
M	1	ORIF Pelvis
M	1	ORIF Right Foot
F	1	ORIF Ankle Left
M	1	ORIF right Acetabelum
F	1	Remove hardware upper limb hand (right) / Ex fix tibial plateau
F	1	ORIF left Radius/Ulna
M	1	ORIF right Tibia
F	1	ORIF left Ankle
F	1	ORIF right Wrist
F	1	ORIF right Femur
M	1	ORIF left Foot
M	1	ORIF left Tibia
F	1	ORIF right Ankle
M	1	ORIF right Foot
F	1	Closed IM Rodding Femur (Bilateral, Leg). ORIF Hip (Right, Hip)
M	1	Closed IM rodding right Tibia
M	2	ORIF left Ankle
M	1	Irrigate & Debride (ORT) (Bilateral, Lower). ORIF right foot
M	2	Closed IM Rodding right Femur
F	2	ORIF right Wrist
F	2	Closed IM rodding right tibia
M	2	ORIF right Foot
F	2	ORIF right Ankle
M	2	ORIF right Tibia
M	2	ORIF left Acetabelum
M	2	ORIF left humerus
M	2	ORIF left Tibia
M	2	ORIF left radius
M	2	ORIF left Radius
M	2	ORIF left Femur
F	2	ORIF left Ankle
F	2	ORIF right Ankle
M	2	Closed IM rodding right femur
M	2	ORIF right Femur
M	2	ORIF right wrist
M	2	ORIF left Ankle
M	2	Ex fix right Tibial Plateau
M	2	Closed IM rodding right tibia
M	2	ORIF left distal femur, ORIF pelvic ring
F	0	ORIF of left tibial plateau
M	1	ORIF right femur
M	2	external fixation of right knee
F	1	ORIF RT Femur
M	1	ORIF right radius/ulna
F	2	ORIF Right Tibia
M	1	Closed reduction right femur shaft, exfix for the right femur, closed reduction right tibia
F	1	removal of external fixator from the right femur
F	2	ORIF RIGHT ANKLE
M	2	ORIF Right Clavicle
M	1	ORIF Tibia
M	1	ORIF R tibia
M	1	ORIF RT ankle
M	1	ORIF left talus
M	2	ORIF right humerus
M	0	ORIF Radius
F	0	ORIF right foot
F	0	ORIF RIGHT talus
M	0	ORIF right radius/ulna
F	0	ORIF right radius/ulna
F	0	ORIF left radius/ulna
M	0	ORIF left clavicle
M	0	Arthroscopy shoulder
M	1	IMN Left acetabulum
M	1	ORIF radius/ulna
M	1	ORIF left clavicle Partial resection of posterior aspect of left ribs
F	1	ORIF left radius/ulna
M	1	ORIF right foot
M	1	ORIF left femur
M	1	ORIF pelvis
M	1	ORIF right foot
M	1	ORIF Right femur
M	1	ORIF right ankle
F	1	ORIF left ankle
F	1	ORIF right ankle
M	1	ORIF right hand
M	1	ORIF right radius
F	1	ORIF tibia
F	1	ORIF left humerus
F	1	ORIF right tibia
F	1	ORIF right humerus
M	1	ORIF clavicle
M	1	ORIF right clavicle
M	1	ORIF pelvis
F	1	ORIF left femur
M	1	ORIF left ulna/radius
F	1	ORIF left tibia
M	1	ORIF radius
F	1	ORIF radius
M	2	ORIF right hand
F	2	ORIF fibula
M	2	ORIF right hand
M	2	ORIF fibula
M	2	ORIF humerus
F	2	ORIF left radius
F	2	ORIF left humerus
M	2	Ex fix pelvis
M	2	ORIF radius/ulna
F	2	ORIF right hand
F	1	ORIF left acetabulum and anterior column
F	2	ORIF left humerus
M	1	ORIF left acetabulum
M	1	Reposition Right Tibia with Internal Fixation Device, Open Approach
F	1	Diagnostic arthroscopy of right knee with partial medial meniscectomy and anterior debridement and debridement of lateral meniscus
F	1	ORIF right radius
F	1	ORIF of a right trimalleolar fracture
F	2	closed reduction percutaneous pinning of extra-articular distal radius fracture
M	0	ORIF Tibia w/ instr
M	1	irrigation debridement with open reduction internal fixation of the first metatarsal
M	2	Right distal femur osteotomy, Hardware removal, graft and malunion correction
F	1	removal of external fixator from the right femur
M	1	Right Femur ORIF
M	1	ORIF right ankle
M	1	BKA
F	1	bilateral SI fracture dislocation who underwent bilateral SI fixation and L5 to ilium and pelvic InFix placement
F	0	ORIF R bimalleolar ankle fracture
F	0	ORIF distal femur
M	1	ORIF LT acetabulum
F	2	Excision of Left Tibia, Open Approach
M	1	Femoroacetabular Impingement Surgery
M	1	Arthroscopy right Knee Surgery
M	1	Arthrodesis Ankle (Left)
F	1	Arthroplasty Hemi Shoulder (Left, Shoulder)
F	2	ORIF ankle w/ instr
F	2	Replacement of other device for musculoskeletal immobilization, Arthrodesis, interphalangeal joint, with internal fixation
F	1	Removal of syndesmotic screws
M	1	Left TKA
F	1	Right THA
M	1	Left TKA
M	2	Left TKA
F	1	Right TKA
F	1	Right TKA
M	1	Right TKA
F	0	Left TKA
F	1	Left THA
F	2	Right TKA
F	1	Left TKA
F	2	Right TKA
F	2	Left TKA
F	2	Right THA
F	2	Left THA
F	2	Left TKA
M	1	Left THA
F	2	Right THA
F	2	Left TKA
F	1	Right TKA
M	1	Left TKA
F	1	Right THA
F	1	Left TKA
F	2	Left TKA
F	1	Right TKA
F	1	Left TKA
M	0	Right THA
M	2	Left TKA
F	1	Right TKA
M	1	Left THA
F	1	Left TKA
F	1	Left THA
F	1	Right TKA
F	1	Left TKA
F	2	Left THA
F	2	Left TKA
F	2	Left TKA
M	2	Right THA
F	2	Bilateral TKA
F	1	Left THA
F	2	Left TKA
F	2	Left TKA
F	2	Right TKA
F	2	Left TKA
F	2	Right TKA
F	1	Right THA
F	1	Right TKA
F	2	Right TKA
F	2	Left TKA
M	1	Right TKA
F	1	Left THA
F	1	Right THA
F	2	Right TKA
M	1	Right TKA
F	1	Right THA
F	1	Right TKA
F	1	Right TKA
M	0	Left THA
F	1	Right THA
M	1	Left TKA
M	0	Left TKA
M	2	Left THA
M	1	Left THA
F	1	Left THA
M	1	Right THA
F	1	Right TKA
M	1	Left TKA
M	1	Right THA
F	1	Right THA
F	2	Right TKA
F	1	Right TKA
F	1	Right TKA revision
F	1	Right TKA
F	1	Left TKA
F	1	Right TKA
F	0	Left THA
F	2	Left TKA
F	1	Left TKA
M	0	Right TKA
F	1	Left TKA
M	2	Right THA
M	1	Right THA
M	2	Right THA
F	1	Right TKA
F	1	Left TKA
F	1	Right TKA w/ Mako
F	1	Right THA
F	0	Left TKA
F	2	Left TKA revision
M	2	Right TKA
F	1	Right TKA w/ Mako
M	2	Left THA
F	2	TKA; ORIF ankle, IM rodding femur
M	1	Left THA
F	1	Right TKA
M	1	Right THA
F	1	Left TKA
F	1	Right THA
F	0	Right THA
F	1	Left THA
F	2	Right TKA
F	2	Right TKA
F	2	Right TKA
F	1	Right THA
F	2	Bilateral TKA
F	1	Right THA
M	2	Right THA
F	1	Right TKA
F	2	Right TKA
F	1	Right TKA
F	1	Left THA
M	1	Left THA
M	1	Left THA
F	2	Right THA
F	1	Left THA
M	1	Left THA
F	2	Right THA
F	1	Right TKA
F	1	Left THA
F	1	Left TKA
F	1	Right TKA
M	1	Right THA
M	1	Right THA
F	2	Bilateral TKA
M	2	Left TKA
F	1	Right TKA
F	0	Left TKA
F	1	Left THA
F	0	Right THA
M	1	Right TKA
F	2	Right TKA
F	1	Left TKA
M	1	Left TKA
F	2	Right TKA
M	1	Right TKA
F	1	Right THA
F	1	Right THA
M	1	Right THA
F	1	Left THA
M	1	Left THA
F	1	Left THA
F	1	Right THA
F	1	Left THA revision
F	1	Left TJA revision
F	2	Left THA
M	2	Left TKA
F	2	Left TKA
F	2	Left TKA revision
M	2	Right TKA revision
F	2	Right TKA
F	2	Right TKA
F	2	Left THA
F	2	Right TKA
F	1	TKA
F	0	Right TKA
F	0	ACDF
M	0	ACDF C5/6, C6/7
M	1	Lumbar fusion w/ instrument. lumbar spinal anthrodesis
M	1	PCDF
M	1	ORIF Clavicle (Right)
F	1	ACDF
F	1	Lumbar Discectomy
M	1	Lumbar fusion L5-S1
F	1	LumboPelvic Fixation
M	1	PCDF
M	1	exploration and scar excision L4-S1 hardware removal fusion decompression L3-S1
F	1	ORIF pelvis. Remove Hardware Spine (lumbar)
F	1	lumbar sacral fixation with bilateral SI screws
M	1	Lumbar decompression
M	1	PCDF, ACDF, cervical arthrodesis
M	1	ACDF
F	1	L4-5, L5-S1 laminectomy
M	1	Lumbar fusion
F	1	Laminectomy Lumbar
M	1	Posterior Lumbar Fusion Posterior w/Instrumentation
M	1	Laminectomy Lumbar
F	1	Laminectomy Lumbar
M	1	Posterior Lumbar Fusion Posterior w/Instrumentation
M	1	ACDF C3-C7
M	1	Fusion Cervical Posterior (Cervical, Anterior, Posterior)
F	1	Fusion Spine Posterior w/ Instrumentation (Lumbar) / L3-S1 decompression with L3-4, L4-5 posterior instrumentation
M	1	ACDF
M	1	Discectomy Lumbar
F	1	ACDF
M	1	Fusion cervical anterior (spine) ACDF
M	1	Lumbar decompression
F	1	Fusion Cervical Anterior
F	1	Fusion Cervical Posterior PCDF
M	1	Fusion Spine Posterior w/ Instrumentation (Lumbar)
F	1	Fusion Spine Posterior w/ Instrumentation (Posterior, Lumbar, Sacral, Spine)
F	1	Lumbosacral decompression
M	1	Laminectomy Lumbar
F	1	Laminectomy Thoracic w/ Fusion (Posterior, Thoracic, Spine)
F	2	L4-5 decompression, discectomy, foraminotomy
F	2	Lumbar laminectomy
M	2	bilateral L5-S1 decompression, posterior and posterolateral foraminotomy of L5 and S1, and discectomy of L5-S1
M	2	Laminectomy T11-12
F	2	Fusion Spine Posterior w/ Instrument
F	2	Lumbar decompression
M	2	ACDF
F	2	ACDF
M	2	Lumbar decompression fusion
F	2	ACDF from C4-6
F	2	Lumbar laminectomy
M	2	ACDF
M	2	PCDF
F	2	ACDF
M	2	Laminectomy Lumbar
F	2	PCDF
F	2	PCDF
F	2	PCDF. Incision & Drainage Spine (Posterior, Spine).
F	2	ACDF
F	2	Lumbar decompression
M	2	L45 decompression
F	2	3-level fusion from 3-4, 4-5, and 5-1
F	2	Discectomy Lumbar
M	2	Lumbar decompression. Closed IM rodding femur (right leg)
F	2	Lumbar decompression L5/ S1
M	2	Laminectomy Lumbar (Right)
M	2	ACDF
M	2	Lumbar Laminectomy
F	2	Laminectomy Lumbar (Posterior, Lumbar, Spine)
F	2	Fusion cervical posterior (cervical)
F	2	lumbar L3-L4 decompression
M	2	Fusion Spine Posterior with Instrumentation
F	2	Laminotomy (hemilaminectomy), with decompression of nerve root(s), including partial facetectomy, foraminotomy and excision of herniated intervertebral disk
F	2	Remove Hardwire Spine (Pelvis)
F	2	Discectomy
F	1	Cervical decompression fusion
F	2	Lumbar decompression fusion
F	2	Decompression Fusion
M	1	Decompression Fusion
F	1	Cervical Decompression Fusion
M	1	Disectomy
M	1	Decompression Fusion
F	1	Decompression Fusion
F	2	Removal of Internal Fixation Device from Lumbar Vertebra
F	2	L5 and S1 Fusion; THA
F	1	Discectomy
F	1	C1-C4 Cervical Decompression Fusion
M	2	lumbopelvic fixation hardware removal
M	1	Anterior Cervical Decompression Fusion
F	1	L1 and L2 fusion
F	1	L2 and L3 Fusion
M	2	Lumbar fusion with hardware
F	1	Rotator cuff repair; ACDF
F	1	Discectomy, hardware removal
M	2	C1-C2 Cervical Decompression Fusion
M	2	spinal fusion
F	1	cervical decompression fusion C1-C4
F	2	lumbar decompression
M	1	lumbar decompression
F	1	lumbar decompression; instrumental ATIO
F	1	lumbar fusion
F	2	cervical decompression fusion
F	2	lumbar decompression
F	2	cervical decompression C2-C7
F	2	lumbar spinal fusion
F	2	lumbar spinal fusion
F	1	Lumbar decompression L34
F	1	PCDF C3-T1; Cervical Decompression Fusion
F	1	Lumbar decompression L5-S1
F	1	Lumbar decompression L5-S1
F	1	Revision C2 decompression
M	2	Cervical decompression fusion; C3-C4 ACDF
F	1	ALIF L5-S1, revision of hardware L4-S1, posterior lumbar decompression L5-S1
M	1	ACDF
F	1	Thoracic/pelvis; T11-L2 with lumbar sacral fixation
F	1	Cervical decompression fusion C3-C4
F	1	L5-S1 Fusion
F	2	Lumber fusion L4-L5
F	1	decompression of L3-L5 revision
F	0	Lumbar Laminectomy
M	2	Iliac screw placement
M	1	Lumbar decompression fusion
F	1	lumbar L2-L3 and L3-L4 decompression with instrumentation and fusion of L3-L4
F	1	ACDF C4-C5, C5-C6
M	1	Cervical Decompression Fusion
M	2	posterior lumbar decompression and instrumentation with posterolateral fusion at L3-L4 with exchange of hardware at the L4-L5
M	1	Lumbar Decompression Fusion
M	2	ACDF
M	2	ACDF
M	1	Lumbar decompression fusion
F	1	Harrington Rod Insertion
M	1	Cervical decompression fusion
F	1	removal of prominent spinal hardware
F	2	Posterior spinal fusion of L5-S1
M	1	Lumbar decompression
F	2	Lumbar decompression L3-4 L4-5
F	2	Excision of intervertebral disc. ACDF
F	1	EXCISION SCAR TISSUE , EXPLORATION SPINAL FUSION L45 L5S1 ,HARDWARE REMOVAL POSTERIOR LUMBAR L4-L5, L5-S1
F	1	bilateral SI fixation. L5 to ilium and pelvic InFix
M	0	Repair achilles tendon (Left)
M	0	Removal of implant; deep (eg, buried wire, pin, screw, metal band, nail, rod or plate)
M	0	Irrigate & Debride (Left, Leg)
F	0	Arthroscopy wrist surgery (Wrist, Right)
F	0	Repair tendon hand/wrist (Finger, Right)
M	0	ORIF right foot. Skin graft
M	1	Irrigate and Debride (ORT) (Right, Leg).
F	1	Remove Hardware Lower Limb (Right, Ankle)
M	1	Irrigate & Debride (ORT) (Bilateral, Lower). ORIF right foot
F	1	Remove hardware lower limb (right, foot)
M	1	Irrigate and Debride (ORT) (Arm, Left)
M	1	Repair Biceps Tendon (Left)
M	1	ORIF right foot. Irrigate & Debride (ORT) (Right, Foot)
M	1	ORIF right lower leg. Graft Skin Split Thickness (PLA) (Right)
M	1	Repair Achilles Tendon
M	1	Resection heterotopic bone (Left)
M	1	ACDF
M	1	Repair right Quadriceps Tendon
F	1	Remove IM Nail w/Insert Antiox Rod (Left, Leg)
F	1	Arthroscopy Knee (Left)
F	1	Contracture of Right Knee. Release Contracture Knee (Knee, Right)
M	1	Arthroscopy Shoulder w/ right Rotator Cuff Repair
F	1	Irrigate and Debride w/ Abx nail (Left, Hip)
M	1	ORIF humerus
F	2	Biopsy bone limb lower (Left)
M	2	Right THA
M	2	Repair of Right Achilles Tendon
M	2	Fracture repair w/ internal fixation
F	2	Remove hardware lower limb (Upper Leg, Left)
F	2	Repair quadriceps tendon (Right)
F	2	Remove hardware lower limb (Ankle)
M	2	ORIF right ankle, Irrigate and Debride right ankle
M	2	Repair quadriceps tendon (Left)
F	2	ACL -Reconstruction
M	1	Reconstruct right Patellofemoral Ligament

Supplemental Table S2

Average MME for each timeframe

14	42	3 months	6 months
0	0	0	0
0	0	0	0
0	0	0	0
0	0	0	0
0	0	0	0
0	0	0	0
0	0	0	0
0	0	0	0
0	0	0	0
0	0	0	0
0	0	0	0
0	0	0	2.3
0	0	0	67.5
0	0	0	5
0	0	0	
0	20	0	
0	5	0	
0	7.5	0	
10	7.5	0	
22.5	30	0	
10	45	0	
5	20	0	
15	15	0	
80	10	0	
15	10	0	
15		0	
6.8		0	
45		0	
5		0	
15		0	
15		0	
15		0	
10		0	
5		0	
22.5		0	
10		20	
20		5	
		15	
		20	
		7.5	
		20	
		10	
		20	
		15	
		15	
		7.5	
		5	
		10	
		51.8	
		20	
		7.5	
		15	
		15	
		80	
		15	
		22.5	
		2.3	
		10	
		67.5	
9.23783783783784	6.8	8.07796610169492	5.34285714285714

Table 1-1 / Hierarchy cluster and K cluster Means tests

Medication	Frequency of	MME	Weeks Post	Medication	Frequency of	MME	Weeks Post
C. Oxycodone	4-6 hours; 3x	33.8	2 weeks	C. Oxycodone	3	33.8	2 weeks
C. Oxycodone	4-6 hours; 3x	33.8	2 weeks	C. Oxycodone	3	33.8	2 weeks
C. Percoet 10mg	As needed 3x	45	3 months	C. Percoet	3	45	3 months
C. Tramadol 50mg	As needed 2x	20	6 months	C. Tramadol	2	20	6 months
C. Norco 5mg	3x	15	3 months	C. Norco 5mg	3	15	3 months
C. Norco 10mg	once a day	10	3 months	C. Norco	1	10	3 months
C. Oxycodone	4-6 hours; 2x	30	2 weeks	C. Oxycodone	2	30	2 weeks
C. Norco 5mg	As needed 1x	5	6 months	C. Norco 5mg	1	5	6 months
C. Percocet 10mg	4-6 hours	30	2 weeks	C. Percocet	2	30	2 weeks
C. Percocet 10mg	4-6 hours	30	2 weeks	C. Percocet	2	30	2 weeks
C. Oxycodone	4-6 hours	45	2 weeks	C. Oxycodone	3	45	2 weeks
C. Tylenol 3	As needed; 1x	2.3	6 months	C. Tylenol 3	1	2.3	6 months
C. Norco 5mg	4-6 hours; 3x	15	2 weeks	C. Norco 5mg	3	15	2 weeks
C. Norco 10mg	As needed; 1x	10	6 months	C. Norco	1	10	6 months
C. norco 10 mg	as needed; 3x	30	2 weeks	C. norco 10	3	30	2 weeks
C. Norco 5mg	2 times/day	20	3 months	C. Norco 5mg	2	20	3 months
C. Norco 10mg	4 hours; 4x	40	2 weeks	C. Norco	4	40	2 weeks
C. Percocet 15mg	as needed; 1x	22.5	6 months	C. Percocet	1	22.5	6 months
C. Oxycodone	As needed; 1x	11.3	6 months	C. Oxycodone	1	11.3	6 months
C. Norco 10mg	as needed; 1x	10	6 months	C. Norco	1	10	6 months
C. Percocet 5mg	once/day	7.5	2 weeks	C. Percocet	1	7.5	2 weeks
C. Percocet 10mg	4 times/day	60	2 weeks	C. Percocet	4	60	2 weeks
C. Tramadol 50mg	2 times/day	10	3 months	C. Tramadol	2	10	3 months
C. norco 5mg	2 times/day	10	2 weeks	C. norco 5mg	2	10	2 weeks
C. Oxycodone	4-6 hours; 3x	45	2 weeks	C. Oxycodone	3	45	2 weeks
C. Norco 7.5mg	as needed; 1x	7.5	6 months	C. Norco	1	7.5	6 months
C. Norco 7.5mg	2 times/day	15	3 weeks (nov	C. Norco	2	15	3 weeks (nov
C. Norco 7.5mg	2 times/day	15	3 months	C. Norco	2	15	3 months
C. Oxycodone	6 hours; 3x	45	2 weeks	C. Oxycodone	3	45	2 weeks
C. norco 10mg	3x	30	2 weeks	C. norco 10mg	3	30	2 weeks
C. Norco 10mg	daily	10	2 weeks	C. Norco	1	10	2 weeks
C. Norco 10mg	2 times/day	20	2 weeks	C. Norco	2	20	2 weeks
C. Norco 10mg	4 times/day	40	3 months	C. Norco	4	40	3 months
C. Tylenol 3	as needed	2.3	6 months	C. Tylenol 3	1	2.3	6 months
C. Oxycodone	4 times/day	60	2 weeks	C. Oxycodone	4	60	2 weeks
C. Percocet 7.5mg	4-6 hours; 4x	45	2 weeks	C. Percocet	4	45	2 weeks
C. Oxxycodone	4 hours; 3x	22.5	2 weeks	C.	3	22.5	2 weeks
C. Percocet 10mg	4-6 hours; 3x	45	2 weeks	C. Percocet	3	45	2 weeks
C. Norco 5mg	2x/day	10	6 months	C. Norco 5mg	2	10	6 months
C. Norco 10mg	2-3x/day	30	2 weeks	C. Norco	3	30	2 weeks
C. Norco 7.5mg	12 hours	7.5	6 months	C. Norco	1	7.5	6 months
C. Norco 10mg	2x	20	2 weeks	C. Norco	2	20	2 weeks
C. Percocet 7.5mg	6 hours; 3x	33.8	2 weeks	C. Percocet	3	33.8	2 weeks
C. Percocet 10mg	4-6 hours; 2x	30	2 weeks	C. Percocet	2	30	2 weeks
C. Percocet 5mg	As needed; 1x	7.5	2 weeks	C. Percocet	1	7.5	2 weeks
C. Tramadol 50mg	as needed; 2x	10	2 weeks	C. Tramadol	2	10	2 weeks
C. Norco 10mg	as needed; 1x	10	3 months	C. Norco	1	10	3 months
C. Tramadol 50mg	as needed; 1x	5	2 weeks	C. Tramadol	1	5	2 weeks
C. Oxycodone	2x	90	6 months	C. Oxycodone	2	90	6 months
Norco 5mg	3x	15	2 weeks	Norco 5mg	3	15	2 weeks
C. Percocet 10mg	As needed; 1x	15	2 weeks	C. Percocet	1	15	2 weeks
C. Percocet 10mg	As needed; 1x	15	2 weeks	C. Percocet	1	15	2 weeks
C. Norco 10mg	1-2x	20	2 weeks	C. Norco	2	20	2 weeks
C. Norco 7.5mg	3x	22.5	2 weeks	C. Norco	3	22.5	2 weeks
C. Norco 10mg	2 times/day	20	3 months	C. Norco	2	20	3 months
C. Norco 7.5mg	4-6 hours; 2x	15	2 weeks	C. Norco	2	15	2 weeks
C. Norco 7.5mg	as needed 2x	22.77	6 months	C. Norco	2	22.77	6 months
C. Norco 10mg	4x	40	2 weeks	C. Norco	4	40	2 weeks
norco 5mg	12 hours 2x	10	2 weeks	norco 5mg	2	10	2 weeks
C. Norco 10mg	as needed; 1x	10	6 months	C. Norco	1	10	6 months
C. Oxycodone	4-6 hours; 2x	90	2 weeks	C. Oxycodone	2	90	2 weeks
C. Tramadol 50mg	as needed; 1x	15	6 months	C. Tramadol	1	15	6 months
C. Percocet 10mg	4-6 hours; 3x	45	2 weeks	C. Percocet	3	45	2 weeks
C. Norco 10mg	4-6 hours; 3x	30	2 weeks	C. Norco	3	30	2 weeks
C. Norco 10mg	4-6 hours; 4x	40	2 weeks	C. Norco 10mg	4	40	2 weeks
D. Morphine 20mg; Oxycontin	as needed; 2x	135	2 weeks	D. Morphine 20mg;	2	135	2 weeks
C. Oxycodone 30mg	4-6 hours; 2x	90	2 weeks	C. Oxycodone 30mg	2	90	2 weeks
C. Norco 10mg	as needed; 4x	40	2 weeks	C. Norco 10mg	4	40	2 weeks
C. Norco 7.5mg	as needed; 2x	15	3 months	C. Norco 7.5mg	2	15	3 months
C. Norco 5mg	3x	15	2 weeks	C. Norco 5mg	3	15	2 weeks
C. Norco 10mg	4x	40	2 weeks	C. Norco 10mg	4	40	2 weeks
C. Percocet 10mg	3x	45	2 weeks	C. Percocet 10mg	3	45	2 weeks
C. Percocet 10mg	3x	45	2 weeks	C. Percocet 10mg	3	45	2 weeks
C. Oxycodone 10mg	4 times/day	45	2 weeks	C. Oxycodone 10mg	4	45	2 weeks
C. Norco 7.5mg	4x	37.5	2 weeks	C. Norco	4	37.5	2 weeks
C. Percocet 7.5mg	4-6 hours	90	2 weeks	C. Percocet 7.5mg	5	90	2 weeks
C. Tramadol 50mg	daily	10	2 weeks	C. Tramadol 50mg	1	10	2 weeks
C. Norco 7.5mg	4-6 hours; 3x	22.5	2 weeks	C. Norco 7.5mg	3	22.5	2 weeks
C. Norco 10mg	3x	30	2 weeks	C. Norco 10mg	3	30	2 weeks
D. Methadone 5mg	2x	80	3 months	D. Methadone 5m	2	80	3 months
C. Norco 10mg	2x	20	3 months	C. Norco 10mg	2	20	3 months
C. Oxycodone 15mg	3x	67.5	2 weeks	C. Oxycodone 15m	3	67.5	2 weeks
C. Percocet 10mg	2x	30	2 weeks	C. Percocet 10mg	2	30	2 weeks
C. Tramadol 50mg	As needed;	5	6 months	C. Tramadol	1	5	6 months
C. Norco 7.5mg	2x	15	2 weeks	C. Norco 7.5mg	2	15	2 weeks
C. Oxycodone 10mg	2x	30	2 weeks	C. Oxycodone 10mg	2	30	2 weeks
Norco 10mg	2x	20	3 months	Norco 10mg	2	20	3 months
Norco 10mg	2x	20	6 weeks	Norco 10mg	2	20	6 weeks
Norco 5mg	1x	5	3 months	Norco 5mg	1	5	3 months
Tylenol 4 15mg	1x	4.5	6 months	Tylenol 4 15mg	1	4.5	6 months
Norco 7.5	2x	15	3 months	Norco 7.5	2	15	3 months
Norco 10mg	2x	20	3 months	Norco 10mg	2	20	3 months
Norco 5mg	1x	5	6 weeks	Norco 5mg	1	5	6 weeks
Norco 7.5	1x	7.5	3 months	Norco 7.5	1	7.5	3 months
Norco 10mg	2x	20	3 months	Norco 10mg	2	20	3 months
Norco 5mg	2x	10	3 months	Norco 5mg	2	10	3 months
Norco 10mg	2x	20	3 months	Norco 10mg	2	20	3 months
Norco 5mg	3x	15	3 months	Norco 5mg	3	15	3 months
Norco 7.5mg	2x	15	3 months	Norco 7.5mg	2	15	3 months
Norco 7.5mg	1x	7.5	3 months	Norco 7.5mg	1	7.5	3 months
Norco 5mg	1x	5	3 months	Norco 5mg	1	5	3 months
Norco 5mg	2x	10	2 weeks	Norco 5mg	2	10	2 weeks
Norco 10mg	1x	10	3 months	Norco 10mg	1	10	3 months
Tylenol 3 15mg	2x	51.8	3 months	Tylenol 3 15mg	2	51.8	3 months
Norco 10mg	2x	20	3 months	Norco 10mg	2	20	3 months
Norco 7.5mg	1x	7.5	3 months	Norco 7.5mg	1	7.5	3 months
Oxycodone 5mg	2x	15	3 months	Oxycodone 5mg	2	15	3 months
Tylenol 3 15mg	1x	2.3	6 months	Tylenol 3 15mg	1	2.3	6 months
Oxycodone 30mg	1.5x 3+months	67.5	6 months	Oxycodone 30mg	1	67.5	6 months
Norco 5mg	3x	15	3 months	Norco 5mg	3	15	3 months
Norco 7.5mg	1x	7.5	6 weeks	Norco 7.5mg	1	7.5	6 weeks
Methadone 10mg	6 tab	80	3 months	Methadone 10mg	3	80	3 months
Norco 5 mg	1x	5	6 months	Norco 5 mg	1	5	6 months
Percocet 5mg	1x	15	3 months	Percocet 5mg	1	15	3 months
Percocet 5mg	3x	22.5	2 weeks	Percocet 5mg	3	22.5	2 weeks
Norco 7.5mg	1x	7.5	6 weeks	Norco 7.5mg	1	7.5	6 weeks
Norco 5mg	2x	10	2 weeks	Norco 5mg	2	10	2 weeks
Norco 10mg	3x 3+months	30	6 weeks	Norco 10mg	3	30	6 weeks
Norco 7.5mg	3x	22.5	3 months	Norco 7.5mg	3	22.5	3 months
Norco 5mg	1x	5	2 weeks	Norco 5mg	1	5	2 weeks
Oxycontin 60mg	12h	90	6 months	Oxycontin	1	90	6 months
Norco 7.5mg	2x	15	2 weeks	Norco 7.5mg	2	15	2 weeks
Oxycodone 15mg	12h 3+ months	45	6 weeks	Oxycodone 15mg	2	45	6 weeks
Methadone 10mg	4 tab	80	2 weeks	Methadone 10mg	1	80	2 weeks
Oxycodone 5mg	2x	15	2 weeks	Oxycodone	2	15	2 weeks
Norco 7.5mg	2x	15	2 weeks	Norco 7.5mg	2	15	2 weeks
Tylenol 3 15mg	3x	6.8	2 weeks	Tylenol 3 15mg	3	6.8	2 weeks
Norco 7.5mg	1x 3+months	15	6 months	Norco 7.5mg	1	15	6 months
Norco 10mg	2x	20	6 weeks	Norco 10mg	2	20	6 weeks
Percocet 10mg	3x 3+months	45	2 weeks	Percocet 10mg	3	45	2 weeks
Norco 5mg	1x	5	2 weeks	Norco 5mg	1	5	2 weeks
Norco 5mg	3x	15	6 weeks	Norco 5mg	3	15	6 weeks
Tylenol 3 15mg	1x	2.3	3 months	Tylenol 3 15mg	1	2.3	3 months
Tramadol 50mg	3x	15	2 weeks	Tramadol 50mg	3	15	2 weeks
Tramadol 50mg	2x	10	3 months	Tramadol 50mg	2	10	3 months
Norco 5mg	3x	15	2 weeks	Norco 5mg	3	15	2 weeks
Percocet 5 mg	3.5x 3+months	26.3	6 months	Percocet 5 mg	3	26.3	6 months
Norco 5mg	3x	15	2 weeks	Norco 5mg	3	15	2 weeks
Tramadol 50mg	2x	10	6 weeks	Tramadol 50mg	2	10	6 weeks
Norco 5mg	2x	10	2 weeks	Norco 5mg	2	10	2 weeks
Norco 5mg	1x	5	2 weeks	Norco 5mg	1	5	2 weeks
Norco 7.5mg	3x	22.5	2 weeks	Norco 7.5mg	3	22.5	2 weeks
Norco 5mg	2x	10	6 weeks	Norco 5mg	2	10	6 weeks
Norco 5mg	2x	10	2 weeks	Norco 5mg	2	10	2 weeks
Oxycodone 15mg	3x	67.5	3 months	Oxycodone 15mg	3	67.5	3 months
Norco 10mg	2x	20	2 weeks	Norco 10mg	2	20	2 weeks
C. Norco 7.5mg	1x	7.5	6 months	C. Norco 7.5mg	1	7.5	6 months
C. Tylenol 3 15mg	1x	2.3	6 months	C. Tylenol 3	1	2.3	6 months
C. Tramadol 50mg	1x	5	2 weeks	C. Tramadol 50mg	1	5	2 weeks
C. Percocet 10mg	2x; 3+ months	45	3 months	C. Percocet	2	45	3 months
C. Norco 5mg	1x	5	6 weeks	C. Norco 5mg	1	5	6 weeks
C. Norco 10mg	2x	10	3 months	C. Norco 10mg	2	10	3 months
C. Oxycodone 30mg	2x 3+months	90	6 months	C. Oxycodone 30m	2	90	6 months
C. Norco 10mg	3x	30	3 months	C. Norco 10mg	3	30	3 months
C. Norco 7.5mg	3x 3+ months	30	3 months	C. Norco 7.5mg	3	30	3 months
C. Norco 7.5mg	3x 3+ months	30	6 months	C. Norco 7.5mg	3	30	6 months
C. Norco 7.5mg	3x 3+ months	30	3 months	C. Norco 7.5mg	3	30	3 months
C. Percocet 5mg	3x 3+ months	30	3 months	C. Percocet 5mg	3	30	3 months
C. Percocet 10mg	3x 3+months	30	6 months	C. Percocet 10mg	3	30	6 months
C. Norco 10mg	1x	10	3 months	C. Norco 10mg	1	10	3 months
C. Norco 10mg	3x	28.13	2 weeks	C. Norco 10mg	3	28.13	2 weeks
C. Norco 10mg	2x	20	6 weeks	C. Norco 10mg	2	20	6 weeks
B,C. Tylenol 4	2x	4.5	6 months	B,C. Tylenol 4	2	4.5	6 months
C. Norco 10mg	3x	30	6 months	C. Norco 10mg	3	30	6 months
C. Percocet 10mg	3x 3+ months	45	6 months	C. Percocet	3	45	6 months
C. Norco 5mg	4x	20	2 weeks	C. Norco 5mg	4	20	2 weeks
C. Norco 10mg	3x	30	6 months	C. Norco 10mg	3	30	6 months
C. Norco 5mg	3x	15	2 weeks	C. Norco 5mg	3	15	2 weeks
C. Norco 5mg	3x	15	6 months	C. Norco 5mg	3	15	6 months
C. Percocet 7.5mg	2x	22.5	2 weeks	C. Percocet	2	22.5	2 weeks
C. Norco 7.5mg	3x	22.5	2 weeks	C. Norco 7.5mg	3	22.5	2 weeks
C. Norco 7.5mg	3x	22.5	2 weeks	C. Norco 7.5mg	3	22.5	2 weeks
C. Norco 7.5mg	3x 3+ months	28.13	5 months	C. Norco 7.5mg	3	28.13	5 months
C. Norco 7.5mg	4-6 hours; 3x	28.13	3 months	C. Norco	3	28.13	3 months
C. Norco 10mg	3 times/day	30	6 months	C. Norco	3	30	6 months
C. Norco 7.5mg	As needed	7.5	6 months	C. Norco 7.5mg	1	7.5	6 months
C. Norco 10mg	2 times/day	20	6 weeks	C. Norco	2	20	6 weeks
C. Norco 7.5mg	as needed	28.13	6 weeks	C. Norco	1	28.13	6 weeks
C. Percocet 10mg	as needed	45	6 months	C. Percocet	1	45	6 months
C. Percocet 10mg	2 times/day	45	6 months	C. Percocet	2	45	6 months
C. Percocet 10mg	2 times/day	30	6 months	C. Percocet	2	30	6 months
C. Norco 10mg	as needed	30	6 months	C. Norco	1	30	6 months
C. Norco 10mg	3 times/day	30	6 months	C. Norco	3	30	6 months
C. Percocet 10mg	as needed	33.75	3 months	C. Percocet	1	33.75	3 months
C. Percocet 10mg	2x/day	30	6 months	C. Percocet	2	30	6 months
C. Norco 10mg	4 hours; 2x	20	2 weeks	C. Norco	2	20	2 weeks
C. Percocet 5mg	4-6 hours	30	3 months	C. Percocet	3	30	3 months
C. Oxycodone	4-6 hours	37.5	6 months	C. Oxycodone	4	37.5	6 months
C. Tramadol 50mg	1X	5	2 weeks	C. Tramadol	1	5	2 weeks
C. Norco 10mg	3x	30	3 months	C. Norco 10mg	3	30	3 months
C. Percocet 10mg	3x	28.13	2 weeks	C. Percocet	3	28.13	2 weeks
C. Percocet 10mg	2x	30	6 months	C. Percocet	2	30	6 months
C. Tramadol 50mg	2x	10	6 months	C. Tramadol	2	10	6 months
C. percocet 15mg	2x	45	6 months	C. percocet	2	45	6 months
C. Norco 7.5mg	2x	15	3 months	C. Norco	2	15	3 months
C. Norco 7.5mg	1x	7.5	6 months	C. Norco	1	7.5	6 months
C. Codeine 60mg	1x/as needed	9	6 months	C. Codeine	1	9	6 months
C. Norco 10mg	1x	10	6 months	C. Norco	1	10	6 months
C. Percocet 10mg	3x	45	6 months	C. Percocet	3	45	6 months
C. Percocet 7.5mg	1x	7.5	6 months	C. Percocet	1	7.5	6 months
C. Norco 5mg	2x	10	6 months	C. Norco 5mg	2	10	6 months
C. Tramadol 50mg	1x	5	6 months	C. Tramadol	1	5	6 months
E. Dilaudid, 0.5 mg,	Q10 min	120	3 months	E. Dilaudid, 0.5	5	120	3 months
C. Oxycodone 7.5mg	1x	11.3	6 months	C. Oxycodone 7.5m	1	11.3	6 months
C. Norco 10mg	1x	10	6 months	C. Norco 10mg	1	10	6 months
C. Norco 10mg	1x	10	6 months	C. Norco 10mg	1	10	6 months
C. Oxycodone 15mg	2x	45	2 weeks	C. Oxycodone	2	45	2 weeks
C. Norco 7.5mg	2x	15	6 weeks	C. Norco 7.5mg	2	15	6 weeks
C. Tramadol 50mg	1x	5	6 months	C. Tramadol	1	5	6 months
C. Percocet 10mg	2x	30	2 weeks	C. Percocet 10mg	2	30	2 weeks
C. Percocet 7.5mg	1x	22.5	6 months	C. Percocet 7.5mg	1	22.5	6 months
		Average MME				Average	
		25.4173933649289				25.4173933649289	
		STD MME				STD MME	
		21.6747417259672				21.6747417259672	

Legend

Class	MME Range	Color
**C (occasional use short acting narcotics)**	**1-30**	
**D (consistent use short acting narcotics)**	**31-79**	
**E (Long acting drugs/IV use)**	**80-135**	

Vadiya's Pain Scale:			Medication Codes:	
0	No Pain		A	0 Meds
1	Tolerable		B	OTC medications
2	Intolerable		C	Occasional Short-acting narcotics: Norco, Percocet, Vicodin Tylenol #3 codeine, Oxycodone, Tramadol
			D	Consistent Short-acting narcotics: Norco, Percocet, Vicodin, Oxycodone
			E	Long acting narcotics: Oxycontin, Fentenol, MS Contin, Morphine/IV Drugs, Methadone; short-acting breakthrough narcotics

MME for each class

C	D	E
20	33.8	90
15	33.8	90
10	45	135
30	45	90
5	40	90
30	60	80
30	45	80
2.3	45	90
15	40	80
10	60	90
30	45	120
20	45	
22.5	33.8	
11.3	40	
10	45	
7.5	40	
10	40	
10	40	
7.5	45	
15	45	
15	45	
30	37.5	
10	67.5	
20	51.8	
2.3	67.5	
22.5	45	
10	45	
30	67.5	
7.5	45	
20	45	
30	45	
7.5	45	
10	33.75	
10	37.5	
5	45	
15	45	
15	45	
20		
22.5		
20		
15		
22.77		
10		
10		
15		
30		
15		
15		
10		
22.5		
30		
20		
30		
5		
15		
30		
20		
20		
5		
4.5		
15		
20		
5		
7.5		
20		
10		
20		
15		
15		
7.5		
5		
10		
10		
20		
7.5		
15		
2.3		
15		
7.5		
5		
15		
22.5		
7.5		
10		
30		
22.5		
5		
15		
15		
15		
6.8		
30		
20		
5		
15		
2.3		
15		
10		
15		
26.3		
15		
10		
10		
5		
22.5		
10		
10		
20		
7.5		
2.3		
5		
5		
10		
30		
30		
30		
30		
30		
30		
10		
28.13		
20		
4.5		
30		
20		
30		
15		
15		
22.5		
22.5		
22.5		
28.13		
28.13		
30		
7.5		
20		
28.13		
30		
30		
30		
30		
20		
30		
5		
30		
28.13		
30		
10		
15		
7.5		
9		
10		
7.5		
10		
5		
11.3		
10		
10		
15		
5		
30		
22.5		

Average MME for each timeframe

2 weeks	6 weeks	3 months	6 months
0	0	0	0
0	0	0	0
0	0	0	0
0	0	0	0
0	0	0	0
0	0	0	0
0	0	0	0
0	0	0	0
0	0	0	0
0	0	0	0
0	0	0	0
0	0	0	0
0	0	0	0
0	0	0	0
0	0	0	0
0	0	0	0
0	0	0	0
0	0	0	0
0	0	0	0
0	0	0	0
0	0	0	0
0	0	0	0
0	0	0	0
0	0	0	0
0	0	0	0
0	0	0	0
0	0	0	0
0	100	0	0
0	100	0	0
0	100	0	0
0	100	0	0
0	100	0	0
0	100	0	0
0	100	0	0
0	100	0	0
0	100	0	0
0	100	0	0
0	100	0	0
0	100	0	0
0	100	0	0
0	100	0	0
0	100	0	0
0	100	0	0
0		0	0
0		0	0
0		0	0
0		0	0
0		0	0
0		0	0
100		0	0
100		0	0
100		0	0
100		0	0
100		0	0
100		0	0
100		0	0
100		0	0
100		0	0
100		0	0
100		0	0
100		0	0
100		0	0
100		0	0
100		0	0
100		0	0
100		0	0
100		0	0
100		0	0
100		0	0
100		0	0
100		0	0
100		0	0
100		0	0
100		0	0
100		0	0
100		0	0
100		0	0
100		0	0
100		0	0
100		0	0
100		0	0
100		0	0
100		0	0
100		0	0
100		0	0
100		0	0
100		0	0
100		100	0
100		100	0
100		100	0
100		100	0
100		100	0
100		100	0
100		100	0
100		100	0
100		100	0
100		100	0
100		100	0
100		100	0
100		100	0
100		100	0
100		100	0
100		100	0
100		100	0
100		100	0
100		100	0
100		100	0
100		100	0
100		100	0
100		100	0
100		100	0
100		100	0
100		100	0
100		100	0
100		100	0
100		100	0
100		100	0
100		100	0
100		100	0
100		100	0
100		100	0
100		100	0
100		100	0
100		100	0
100		100	0
100		100	0
100		100	100
100		100	100
100		100	100
100		100	100
100		100	100
100		100	100
100		100	100
100		100	100
100		100	100
100		100	100
100		100	100
100		100	100
100			100
100			100
100			100
100			100
100			100
			100
			100
			100
			100
			100
			100
			100
			100
			100
			100
			100
			100
			100
			100
			100
			100
			100
			100
			100
			100
			100
			100
			100
			100
			100
			100
			100
			100
			100
			100
			100
			100
			100
			100
			100
			100
			100
			100
			100
			100
			100
